# Altered T-Cell Function in Schizophrenia: A Cellular Model to Investigate Molecular Disease Mechanisms

**DOI:** 10.1371/journal.pone.0000692

**Published:** 2007-08-01

**Authors:** Rachel M. Craddock, Helen E. Lockstone, David A. Rider, Matthew T. Wayland, Laura J.W. Harris, Peter J. McKenna, Sabine Bahn

**Affiliations:** 1 Institute of Biotechnology, University of Cambridge, Cambridge, United Kingdom; 2 Institute of Molecular and Cell Biology, Proteos, Singapore; 3 Fulbourn Hospital, Cambridge, United Kingdom; New York University School of Medicine, United States of America

## Abstract

Despite decades of research into the aetiology and pathophysiology of schizophrenia, our understanding of this devastating disorder remains incomplete, with adverse consequences for both diagnosis and treatment. Here we investigate whether differences between patients and controls can be observed in peripheral patient tissue, with a view of establishing a means for dynamic investigations into cell function. *In vitro* stimulation of peripheral blood CD3+ pan T cells with anti-CD3 (clone OKT3) was used to investigate disease-associated cell responses. T cells from both medicated (n = 39), unmedicated (n = 6) and minimally medicated (n = 5) schizophrenia patients were found to have significantly lower proliferative responses to stimulation, compared to well-matched controls (n = 32). Expression of CD3 and TCR (T cell receptor) αβ chains was equivalent between patients and controls, ensuring equal stimulation with anti-CD3, and there was no significant difference in the proportions of CD4+ and CD8+ T cells between samples (n = 12). Lower T cell proliferation in schizophrenia patients was not found to result from deficient early tyrosine phosphorylation signalling or lower IL-2 (interleukin-2) production, as these parameters were similar between patients and controls, as was the expression of CD25, the IL-2 receptor α chain. Analysis of CD45 isoforms, however, revealed that patients had a significantly greater percentage of CD8+ and CD4+ CD45RA+ cells before stimulation and significantly higher fluorescence intensity of CD45RA on CD4+ and CD8+ cells before and after stimulation. There was significantly higher expression of CD45 RB on both CD4+ and CD8+ unstimulated cells, with a trend towards lower numbers of CD45RO+ T cells in patient blood. Gene expression analysis in freshly isolated T cells from six minimally treated or first onset patients and six controls was carried out using human whole-genome CodeLink microarrays to identify functional pathways that may affect the ability of patient cells to respond to stimulation. Functional profiling showed prominent transcript changes in categories pertaining to cell cycle machinery, intracellular signalling, oxidative stress and metabolism. Intriguingly, chromosomal location analysis of genes significantly altered between schizophrenia and controls revealed clusters at 1p36, 1q42 and 6p22, which have previously been identified as strong susceptibility loci for schizophrenia.

## Introduction

Effective diagnosis and treatment of schizophrenia remains an enormous problem for patients and clinicians while the aetiology of the disorder and the underlying pathophysiological mechanisms are not fully understood. The very nature of this disorder makes it difficult to find suitable research tools and accessible tissues for experimentation, especially as it remains unclear whether pathological differences in schizophrenia can be detected outside the brain. Many studies carried out to date have focused on human post mortem brain tissue and unfortunately, problems including drug treatment and post mortem effects can impede the acquisition of high quality data, masking important disease-related changes. The key aim of the current study was therefore to establish a suitable surrogate cell system, free from the influence of post mortem artefact, in which to investigate dynamic functional investigations of disease-associated pathophysiological mechanisms as well as the identification of schizophrenia biomarkers, whilst limiting problems such as drug effects.

In the present study, peripheral blood T cells were utilised to perform dynamic investigations into cell function using *in vitro* stimulation. T cells are a promising candidate for investigations into cellular function as *in vitro* stimulation allows for thorough examination of a variety of key cellular mechanisms, including intracellular signalling and gene transcription. This system makes it possible to identify any subtle deficiencies in systemic cell function which may underpin some of the clinical characteristics of this disorder. Stimulation of T cells can be carried out *in vitro* by mimicking a T cell receptor (TCR) signal via cross-linking of cell surface CD3, using a monoclonal antibody. This ultimately results in cell cycle entry and production of cytokines, particularly IL-2 which is used by T cells in an autocrine fashion to drive proliferation. There is also up regulation of various activation markers, including CD25, which is the specific α-chain subunit of IL-2, facilitating T cell responses to this cytokine. As T cell activation requires receptor signalling, transcription factor activation, gene transcription, protein synthesis and protein trafficking, systemic abnormalities in these physiological processes in schizophrenia can potentially be traced in this model by using downstream effects of stimulation such as proliferation, cytokine production and gene transcription as readouts of cell function.

## Materials and Methods

### Sample collection

Peripheral blood was taken from well characterised medicated schizophrenia patients who met DSM-IV criteria for a diagnosis of schizophrenia, and minimally medicated patients with a confirmed diagnosis of schizophrenia who had either received less than 4 weeks of therapy, or who were non-compliant with drug therapy. Blood was also taken from unmedicated patients with first episode psychosis, who presented with clinical symptoms consistent with a diagnosis of schizophrenia (DSM-IV). Additionally, the majority of patients in this study were assessed by a standardised interview (SCID) and the Positive and Negative Symptoms Scale (PANSS). For each patient sample, blood was taken from corresponding age, sex, and race matched controls. Controls were also matched as far as possible for smoking. Patients and controls were excluded from the study if they had any co-morbidity such as diabetes, heart disease, thyroid disease, autoimmune disease or any recent infections and patients with a history of substance abuse were excluded. All patient samples were processed concomitantly with their respective controls (see patient demographics, Table 1). Written consent was obtained from subjects in line with Cambridgeshire Local Ethics Committee approval.

**Table 1:** Demographic details of patients and controls

		Proliferation Medicated		Minimally medicated		Microarrays		Flow cytometry	
		Control	Patient	Control	Patient	Control	Patient	Control	Patient
Age		34±10.5	35±10.8	29±6.9	28±10.4	30±6.4	31±14.1	31±7.3	33.2±14.3
Sex	Male	21	35	10	9	3	2	9	9
	Female	11	4	2	2	3	4	7	4
Race	White	26	35	7	7	6	6	16	13
	Black	1	3	1	1	-	-	-	-
	Asian	4	1	4	3	-	-	-	-
	Oriental	1	-	-	-	-	-	-	-
Smoking	Smoker	12	20	2	4	2	4	7	6
	Non-Smoker	15	7	10	5	4	2	9	7
	Not known	5	12	-	2	-	-	-	-

Age, sex and race matched controls were compared to medicated schizophrenia patients and a set of minimally medicated and unmedicated schizophrenia patients for proliferation assays. T cells from minimally medicated and unmedicated patients were also used to assess differential gene expression by microarray and for flow cytometric analysis of cell surface marker expression. Ages are expressed as mean ± standard deviation. Patients with recent substance abuse were excluded from the study.

### T cell isolation

CD3+ T cells were isolated from the peripheral blood of schizophrenia patients and age, sex and race-matched controls. In brief, peripheral blood was taken using the S-monovette blood collection system containing EDTA (Sarstedt). Mononuclear cells (PBMC) were isolated by centrifugation over Ficoll-Paque (Amersham Biosciences, Amersham, UK) and CD3+ pan T cells were then purified from these by negative selection using MACS human pan T cell isolation kit with LS separation columns (Miltenyi Biotech, UK). T cell purity was routinely above 98%, when analysed for CD3-ε expression by flow cytometry (FACS Calibur, Becton Dickinson). Where indicated, cells were cultured at 37°C in RPMI medium containing 10% foetal bovine serum and 1% penicillin, streptomycin and glutamine (Sigma, UK).

### T cell proliferation

Proliferative responses to stimulation were measured using ^3^H-thymidine incorporation into progeny cell DNA. T cells were cultured for 48 hours in 96 well plates coated with 0 µg/ml, 0.01 µg/ml, 0.1 µg/ml and 1 µg/ml anti-CD3 (clone OKT3), seeded at a density of 2×10^5^ cells / well to stimulate entry into the cell cycle. Cells were pulsed with of 0.037MBq (1 µCi) ^3^H-thymidine (Amersham Biosciences, UK) per well for a further 24 hours to allow incorporation into DNA and harvested onto 96 well filter plates (Perkin Elmer) to capture labelled DNA. Labelled DNA and hence proliferation was measured using a scintillation counter (Top Count, Packard). All conditions were carried out in triplicate and statistical significance was determined using a non-parametric Mann-Whitney *U* test, with a p-value of less than 0.05 considered significant.

### Analysis of cell surface markers by flow cytometry

Patient and control T cells were cultured for 72 hours in the presence or absence of 1 µg/ml plate-bound anti-CD3. Cells were counted and 5×10^5^ cells/sample were washed in FACS buffer (PBS, 2% foetal bovine serum, Sigma, UK), resuspended and stained in 100 µl FACS buffer (PBS, 2% foetal calf serum, Sigma, UK) containing either anti-CD3 Cy5 (Dako, Denmark)/anti-CD4 FITC (BD Biosciences, Pharmingen, UK)/anti-CD8 PE (Biosciences, Pharmingen, UK), anti-CD4 PE (Biosciences, Pharmingen, UK)/anti-CD25FITC (Immunotech, France), anti-CD4 PE/ anti-CD45RA FITC (Serotec, UK), anti-CD4 PE/ anti-CD45RB FITC (Serotec, UK), anti-CD45RO PE (Serotec, UK) or anti-CD4 FITC/anti-TCRαβ PE (Biosciences, Pharmingen, UK). Cells were incubated at 4°C for 20 minutes before further washing in FACS buffer. Cells were counted using FACS Calibur and Cell Quest software (BD Biosciences, UK). Data were analysed using Flowjo (Treestar, USA). In brief, lymphocytes were gated using forward scatter and side scatter parameter as an indication of cell size and granularity in order to exclude non-cellular debris. The lymphocyte population was analysed according to CD4 and CD8 expression and each parameter was measured separately on CD4+ populations and CD8+ populations. Significance was determined using the Mann-Whitney U Test.

### *Measurement of IL-2 using* Beadlyte® human 22-plex cytokine detection system

T cells were cultured at a density of 0.2×10^5^ cells/well for 48 hours in 96 well plates cultured with 0 µg/ml, 0.01 µg/ml, 0.1 µg/ml and 1 µg/ml anti-CD3 clone OKT3. Supernatants were carefully transferred to microfuge tubes, centrifuged to remove contaminating cells and stored at −80°C until use. Production of IL-2 was measured in T cell supernatants using Beadlyte® human 22-plex cytokine detection system (Upstate, USA), following manufacturers protocol and results were read on a Luminex® 100™ system (Biorad, UK).

### Microarray analysis of T cell gene expression

Differential gene expression between T cells from six minimally treated schizophrenia patients and six age, sex and race matched controls was investigated using CodeLink™ Human Whole Genome Bioarrays (GE Healthcare, UK). Total RNA was extracted from freshly isolated T cells using QIAamp RNA blood mini kit (Qiagen, UK) and quality was assessed with a high-resolution electrophoresis system (Agilent Technologies, Palo Alto, CA, USA). Biotin-labelled cRNA was generated from each sample following the manufacturer's protocol. cRNA was hybridised onto CodeLink whole genome microarray slides, washed and hybridised cRNA species were detected using Cy5-Streptavidin (Ameraham, UK). Slides were scanned using GenePix Personal 4100A Microarray Scanner (Axon Instruments) and analysed with CodeLink Expression Analysis software.

### Preprocessing and normalization of microarray data

Probes were initially filtered to include only those with signal above background noise level, using the strict criterion that probes must be flagged ‘good’ by the CodeLink software on all chips in the experiment. This reduced the number of probes included in the analysis from 53485 to 12416. The spot mean signal intensities for these probes were read into the R statistical program (http://www.r-project.org/) for further analysis, using Bioconductor [1] packages where appropriate. Data were normalised using the quantile method [2] and quality control (QC) procedures were performed to identify potential outlier chips. These included analysis of pairwise correlations of normalised expression values for all chips, boxplots of the normalised expression values for each chip and comparing each chip to a pseudo-median chip. With these QC steps, any chip showing a markedly different profile to others in the experiment can be visually identified; however, there are no defined criteria to help decide whether a chip should be classed as an outlier and excluded. In this study, all chips showed very comparable profiles and hence the final analysis was conducted on 6 schizophrenia and 6 control samples.

### Detection of differentially expressed genes in freshly-isolated T-cells

Schizophrenia patient and control samples were paired according to demographic variables as well as collection and processing of the samples. Paired t-tests between patients (n = 6) and controls (n = 6) were performed using the Limma (linear models for microarray data) package to identify differential expression [3]. A correction for multiple testing was applied with the ‘qvalue’ package [4] and probes with q<0.05 were considered differentially expressed.

### Pathway Analysis

Onto-Express [5], [6] was used to perform functional profiling of the genes significantly altered in freshly-isolated T-cells from schizophrenia patients compared to controls. This analysis identifies gene ontology (GO) categories that are over-represented in the list of significant probes relative to the representation on the array. Significant probe IDs were mapped to corresponding Entrez IDs using Onto-Translate [6] and submitted to Onto-Express. Default settings were used and the reference array was the Amersham CodeLink human whole genome. Biological process categories with a corrected p<0.05 and containing more than 2 genes were selected as the most important pathways affected in the disease state.

### Chromosomal mapping

Chromosomal mapping and analysis was performed using an in-house algorithm (see supplementary information for details). This method was designed for use with Affymetrix data and so CodeLink probe IDs were first mapped to Affymetrix probe-set IDs using Onto-Translate [6]. The main steps in the analysis are outlined below: (i) a representative probe-set for each gene was selected, using alignment score information provided by Affymetrix (www.affymetrix.com). The representative probe-sets were then mapped to their alignment region and discarded if this did not correspond to the chromosomal location of the target gene; (ii) a sliding window of length 5 MB was used to assess the distribution of differentially expressed genes across the genome; (iii) each window was assigned a score, based on the binomial distribution, with high scores corresponding to regions that contain an excess of differentially expressed genes; (iv) based on the scores, chromosomal regions containing a high proportion of differentially expressed genes, which may have some biological significance, were identified.

## Results

### Proliferative responses to stimulation with anti-CD3 are significantly lower in patients with schizophrenia

**Figure 1:**
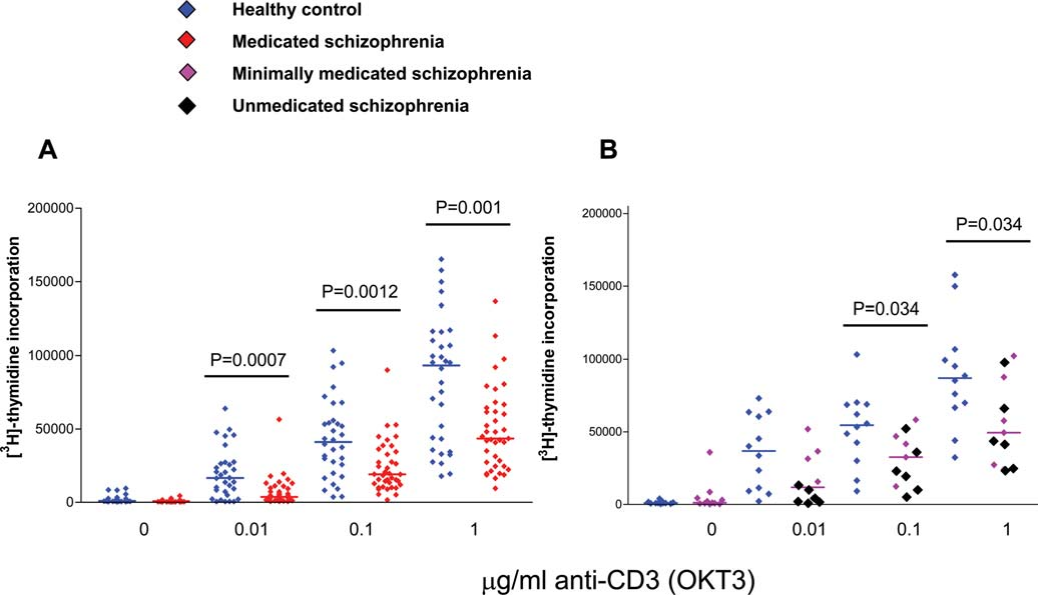
Patients with schizophrenia show lower proliferative responses to stimulation with anti-CD3. (A) Medicated schizophrenia patients were found to have significantly lower proliferative responses to *in vitro* stimulation compared to healthy controls at all concentrations of stimulation with anti-CD3. (B) T cell proliferation was also measured in minimally medicated and unmedicated patients to rule out the possibility of drug effect. These also showed significantly lower proliferative responses to stimulation.

^3^H-thymidine incorporation was used to measure the proliferation of peripheral blood T cells from 39 medicated schizophrenia patients and 32 controls (Figure 1A), stimulated with 0 µg/ml, 0.01 µg/ml, 0.1 µg/ml and 1 µg/ml anti-CD3. Medicated patients with schizophrenia were found to have significantly lower responses to stimulation at all concentrations of anti-CD3 (0.01 µg/ml anti-CD3 p = 0.0007, 0.1 µg/ml anti-CD3 p = 0.001, 1 µg/ml anti-CD3 p = 0.001) when analysed using non-parametric Mann-Whitney U test.

In order to exclude the possibility that lower proliferative responses were a drug effect, changes in unmedicated and minimally medicated individuals were also examined (Figure 1B). ^3^H-thymidine incorporation was measured in 11 minimally and unmedicated patients and 12 matched controls with significantly lower proliferative responses to stimulation with anti-CD3 at concentrations of 0.1 µg/ml (p = 0.034) and 1 µg/ml (p = 0.034).

### Proportions of CD4+ and CD8+ T cells are similar between patients and controls

As CD3+ pan T cells were isolated and used for all experiments, it was necessary to measure the proportions of CD4+ and CD8+ T cells within these populations, as differences in these may also affect proliferation and also make the samples more heterogeneous.

**Figure 2:**
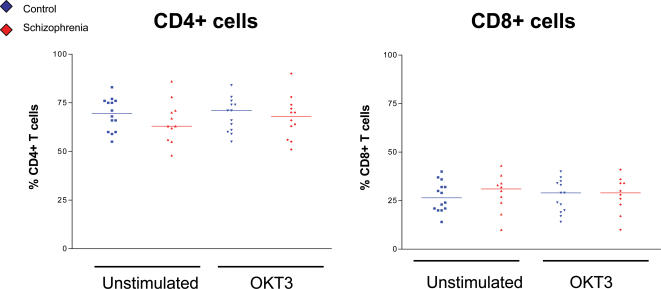
Proportions of CD4+ and CD8+ T cells are similar in schizophrenia patients compared to controls. Expression of CD4 and CD8 was measured on CD3+ T cells from minimally medicated and unmedicated patients and controls. There was no significant difference in proportions of CD4 and CD8 T cells between patients and controls either before of after stimulation, suggesting that there is not preferential expansion of either of these populations following stimulation.

There were no significant differences in the numbers of CD4+ and CD8+ T cells between patient and control samples (Figure 2). Importantly, there was also no significant difference in the percentages of CD4+ and CD8+ cells following stimulation, suggesting that each of these cell subtypes were proliferating proportionally within samples. Populations of CD4+ and CD8+ cells were subsequently analysed separately for expression of CD3, TCRαβ, CD25 and CD45 isoforms.

### Lower proliferative responses in schizophrenia T cells are not a result of lower CD3 or TCR αβ expression

**Figure 3:**
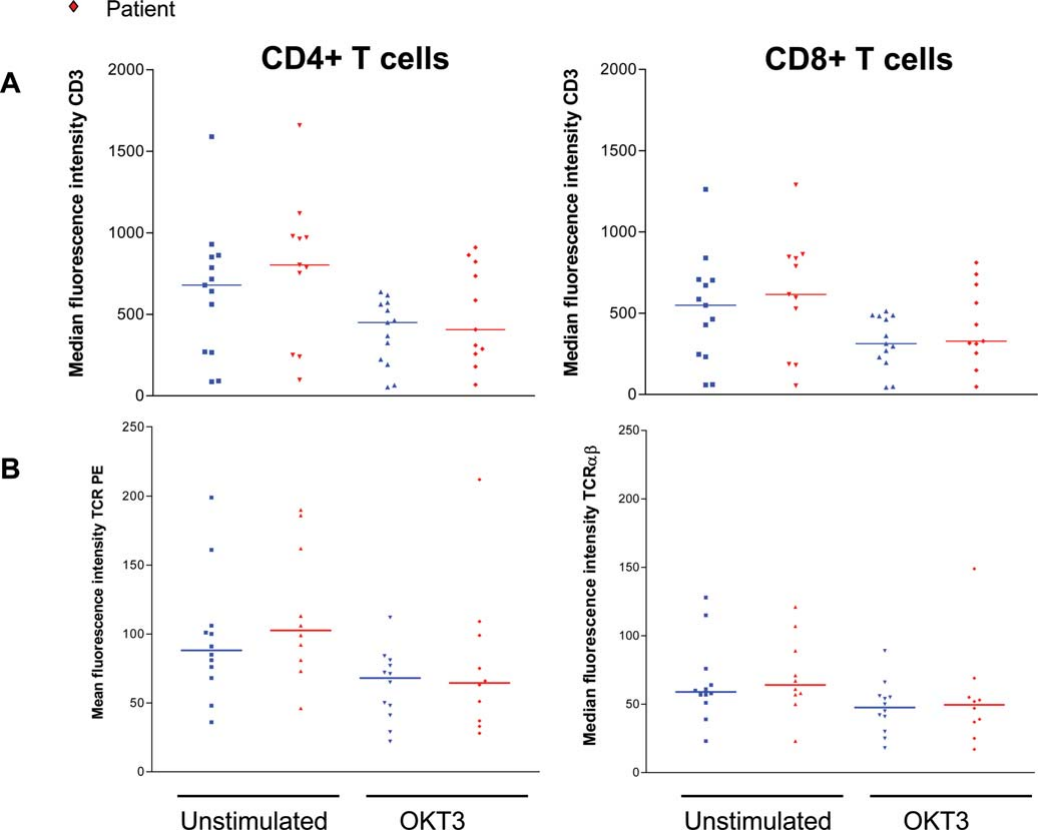
Patient and control T cells show similar levels of CD3 and TCRαβ expression before and after stimulation. (A) CD3 Expression was measured by flow cytometry on patient and control T cells before and after stimulation with anti-CD3. There was no significant difference in the level of expression between patients and controls. (B) Expression of TCR αβ chains on the surface of T cells was also comparable between patients and controls before and after stimulation.

Having observed a lower response to stimulation with anti-CD3 in both treated and untreated patients, we investigated whether this was due to lower surface expression of this molecule on schizophrenia patient T cells. CD3 expression was measured in T cells from patients and controls before and after stimulation using an antibody against CD3ε, conjugated to Cy5 (Figure 3A). There was no significant difference in the expression of CD3 on T cells between patients and controls both in unstimulated cells and in those treated with anti-CD3, indicating that the lower proliferative responses of patients were not a result of lower CD3 expression. Similarly, expression of TCRαβ was compared between patients and controls before and after stimulation (figure 3B). The TCR is down regulated following stimulation as a means of modulating responses and can affect rates of proliferation following stimulation. Patients and controls had comparable levels of TCRαβ in both unstimulated and OKT3-stimulated samples, where the TCR was down regulated to a similar degree.

### Lower proliferative responses in patient T cells is not a result of lower IL-2 production or lower expression of the IL-2 receptor CD25

**Figure 4:**
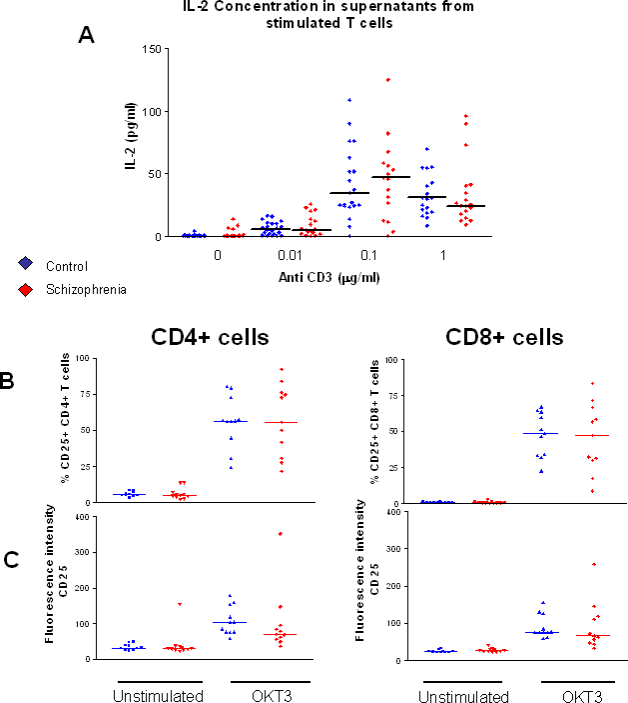
Lower T cell proliferation in schizophrenia does not result from deficient IL-2 production, or receptor expression. (A) IL-2 production was measured in supernatants from patient and control T cells stimulated with 0 µg/ml, 0.01 µg/ml, 0.1 µg/ml and 1 µg/ml OKT3. There was no significant difference in production of IL-2 between patients and controls at each concentration of OKT3. (B) The percentage of CD25+ CD4+ and CD8+ T cells was similar between patients and controls before and after stimulation and there was no significant difference in level of expression of CD25 before or after stimulation (C).

Following stimulation, T cells produce and use IL-2 in an autocrine fashion which is required to drive proliferation [7]. As production and response to this cytokine can both greatly influence rates of proliferation, IL-2 production was compared between patients and controls. IL-2 production was measured in supernatants from OKT3-stimulated and unstimulated T cell samples using a Beadlyte® human 22-plex cytokine detection system. There was no significant difference in the production of IL-2 by patient and control T cells stimulated with plate-bound anti-CD3 at concentrations of 0 µg/ml, 0.01 µg/ml, 0.1 µg/ml and 1 µg/ml (figure 4A), demonstrating that insufficient IL-2 production is not responsible for lower T cell proliferative responses observed in schizophrenia patients.

Expression of CD25 (the IL-2 receptor alpha chain) on CD4+ and CD8+ T cells was also investigated using flow cytometry to investigate whether patient T cells are unable to respond to IL-2 as efficiently as healthy controls. CD25 can also be used as a marker for T cell activation as it is not generally expressed on unstimulated cells, but is up regulated in response to stimulation [8]. There was no significant difference in the percentage of cells expressing CD25 before or after stimulation in patients compared to controls (Figure 4B), demonstrating that patient cells are able to up regulate expression of this receptor, allowing them to respond to IL-2. Similarly, the level of expression of CD25 on patient and control cells before and after stimulation were comparable (Figure 4C).

### Schizophrenia patients have higher numbers of circulating CD45RA+ and CD45RB+ T cells

CD45 is a transmembrane protein tyrosine phosphatase that plays an essential role in early activation of tyrosine kinases necessary for TCR signal transduction. It is composed of large heavily glycosylated extracellular domains that can exist in at least eight different isoforms due to the process of alternative RNA splicing of the extracellular domains [9]. The expression of combinations of extracellular domains results in the isoforms CD45RA, CD45RB, CD45RC and CD45RO, which can be identified using specific monoclonal antibodies. Differential expression of these isoforms can be used as an estimation of T cell activation state from antigen exposure [10]. Naïve cells express the high molecular weight CD45RA and activated and memory cells express the low molecular weight CD45RO [11]. CD45RB is expressed on intermediates as cells differentiate into memory cells.

**Figure 5:**
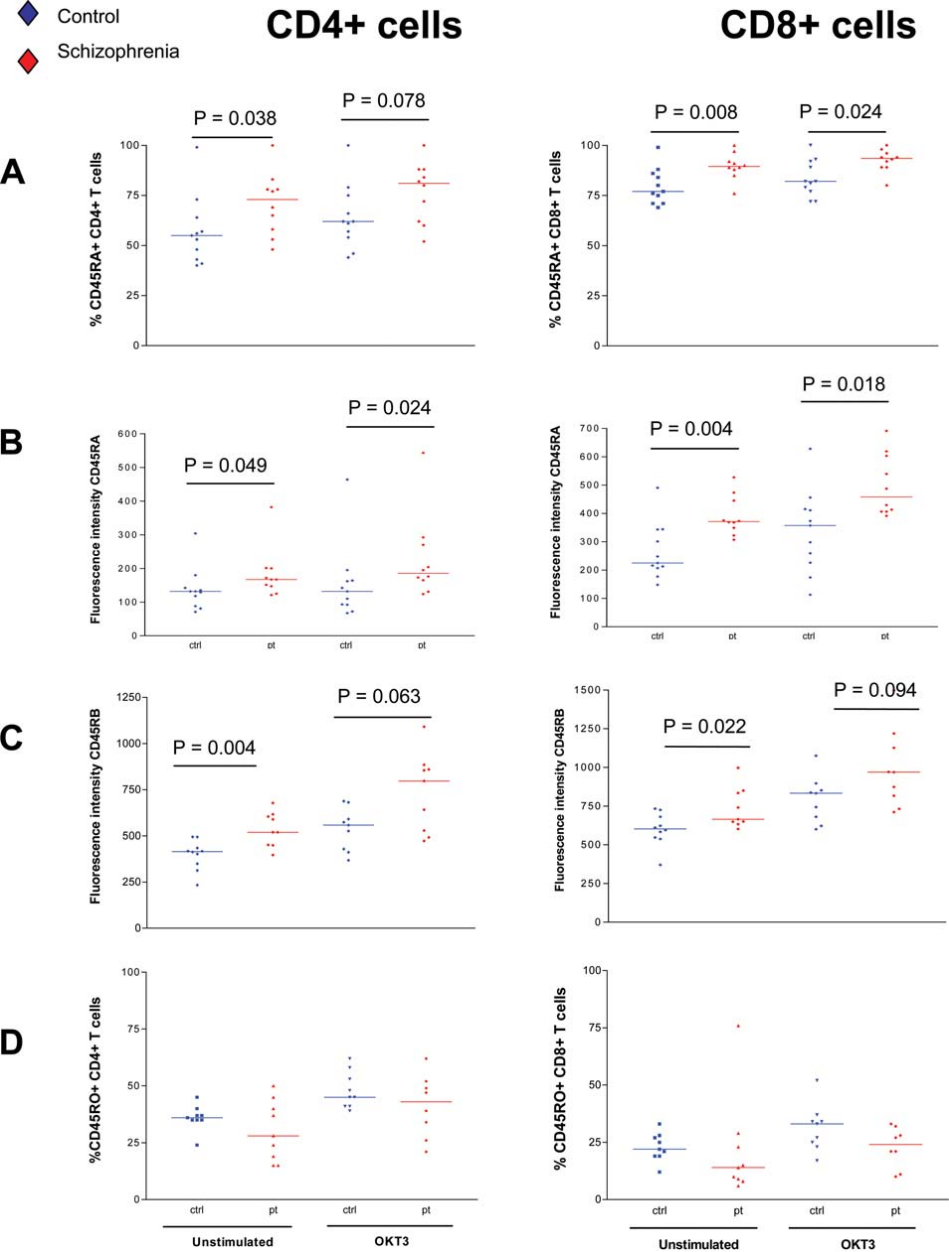
Schizophrenia patients have higher proportions of circulating CD45RA+ and CD45RB+ T cells. Expression of CD45RA, CD45RB and CD45RO was measured on CD4+ and CD8+ T cells from patients and controls in order to assess activation state with regard to antigen experience. (A) The percentage of CD45RA+ cells was significantly higher in patients compared to controls before stimulation and the level of expression of CD45RA was also higher on patient CD4+ and CD8+ cells before and after stimulation (B). (C) CD45RB expression was significantly higher on CD4+ and CD8+ patient T cells before stimulation. (D) There was no significant difference in the expression of CD45RO on either CD4+ or CD8+ T cells from patient or control cells.

Expression of CD45RA, CD45RB and CD45RO isoforms was measured on T cells before and after stimulation in order to assess differences in T cell sub-populations between patients and controls. CD45RA was expressed on a significantly higher percentage of patient CD4 and CD8 T cells before stimulation (figure 5A: % CD45RA+ unstimulated CD4 cells p = 0.038, % CD45RA+ unstimulated CD8 cells p = 0.008) and expression intensity, proportional to the amount of receptor expressed on the cell, was significantly higher in patient CD4 and CD8 cells both before and after stimulation (Figure 5B: Fluorescence intensity CD45RA before stimulation on CD4+ cells p = 0.049 and after stimulation p = 0.024, fluorescence intensity of CD45RA on CD8 cells before stimulation p = 0.004 and after stimulation p  =  0.018). CD45RB was expressed on 100% of patient and control cells before and after stimulation (data not shown) and expression intensity was found to be significantly higher on CD4 and CD8 patient cells before stimulation (Figure 5C: Fluorescence intensity of CD45RB before stimulation on CD4+ cells p = 0.004 and on CD8+ cells p = 0.022 ).

The percentage of CD45RO+ patient and control T cells was also measured before and after stimulation and although differences were not significant, there was a trend towards lower numbers of CD45RO+ patient T cells (Figure 5D).

### Analysis of differentially expressed genes in freshly isolated T cells from schizophrenia patients and matched controls

T cell proliferative responses to stimulation with anti-CD3 can be influenced by many factors. The processes involved in T cell activation include cell signalling, gene transcription, protein synthesis and trafficking, entry into cell cycle and cytokine secretion. Dysfunction in any of these may result in the observed lower proliferative responses of patients. Preliminary studies were initially conducted to investigate early signalling, 5 minutes after T cell stimulation with anti-CD3. This was carried out by Western blot analysis on T cell lysates using an antibody raised against global phospho-tyrosine. No differences were evident in the patterns of tyrosine phosphorylation between patients and controls (data not shown), suggesting that deficits responsible for the lower proliferative responses lie further downstream of events following T cell stimulation.

We employed CodeLink™ Human Whole Genome microarrays to profile gene expression in peripheral blood T cells from unmedicated and minimally medicated patients (n = 6) and controls (n = 6), in order to identify altered gene expression that could underlie lower proliferative responses in patients. Paired t-tests were then used to identify significantly differentially expressed genes, resulting in 399 probes significant at q<0.05 after multiple testing correction. Of these, 320 (80%) probes were decreased in schizophrenia and 79 were increased.

### Functional profiling of significantly altered transcripts

OntoExpress [5], [6] was used to assign functional categories to the significantly altered genes and to identify processes that were over-represented in the list of significant genes. This analysis revealed five significant categories pertaining to cell cycle, including cell cycle (p = 0.0005), cell cycle arrest (p = 0.0007), negative regulation of cell cycle (p = 0.001), mitosis (p = 0.005) and regulation of cell cycle (p = 0.039) (Table 2). These categories each contained a combination of up- and down regulated transcripts. It is intriguing that categories pertaining to cell cycle were significantly altered in freshly isolated, unstimulated T cells in light of the lower proliferative responses to stimulation with anti-CD3 observed in patients.

**Table 2:** Functional pathways associated with cell cycle, cell signalling and oxidative stress and metabolism were significantly altered in schizophrenia by microarray analysis of gene expression.

CELL CYCLE					
	GO ID	Category	Number significant genes in category	Total number genes in category	Corrected P-Value
	GO:0007049	cell cycle	8	307	4.878E-04
	GO:0007050	cell cycle arrest	4	83	7.352E-04
	GO:0045786	negative regulation of cell cycle	4	107	1.112E-03
	GO:0007067	mitosis	3	106	5.257E-03
	GO:0051301	cell division	3	158	1.224E-02
	GO:0000074	regulation of cell cycle	3	283	3.859E-02
CELL SIGNALLING					
	GO:0007242	intracellular signaling cascade	4	451	0.0495
	GO:0006468	proteinamino acid phosphorylation	7	628	0.0153
	GO:0007267	cell-cell signaling	5	331	0.0116
	GO:0007165	signal transduction	18	1503	0.0009
METABOLISM AND OXIDATIVE STRESS					
	GO:0006979	response to oxidative stress	4	57	2.725E-04
	GO:0008152	metabolism	6	481	1.313E-02
	GO:0006118	electron transport	8	390	9.889E-04

Onto-Express was used for pathway analysis of significantly altered genes by identifying gene ontology (GO) categories that are over-represented in the list of significant genes relative to the representation on the array. Five categories associated with cell cycle were found to be significantly involved, including cell cycle (p = 0.0005), cell cycle arrest (p = 0.0007), negative regulation of cell cycle (p = 0.001), mitosis (p = 0.005) and regulation of cell cycle (p  =  0.039).

Four categories associated with cell signalling were found to be significantly altered, including signal transduction (p = 0.0009), cell-cell signalling (p = 0.012), protein amino acid phosphorylation (p = 0.015) and intracellular signalling cascade (p = 0.050).

Three categories involved in oxidative stress were found to be significantly altered, including response to oxidative stress (p = 0.0003), electron transport (p = 0.001) and metabolism (p = 0.013).

Four categories relating to intracellular signalling were also significantly different between patients and controls. Abnormalities with signalling pathways potentially hold considerable consequences for proliferative responses to anti-CD3. The four pathways pertaining to cell signalling were signal transduction (p = 0.0009), cell-cell signalling (p = 0.012), protein amino acid phosphorylation (p = 0.015) and intracellular signalling cascade (p = 0.050).

Other categories that were revealed as significantly altered by OntoExpress included response to oxidative stress (p = 0.0003), electron transport (p = 0.001) and metabolism (p = 0.013).

### Chromosomal location of significantly altered transcripts

**Figure 6:**
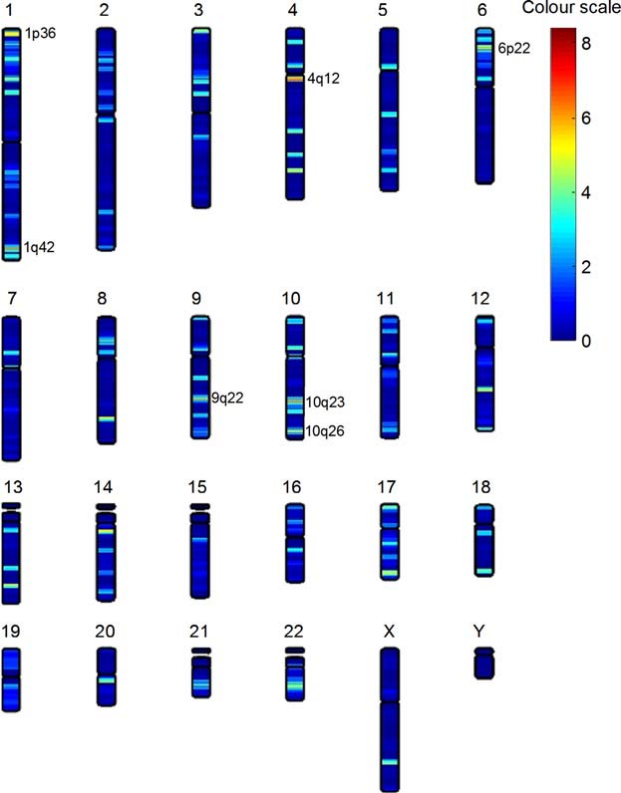
Genes significantly altered between patient and control T cells map to known schizophrenia susceptibility loci. A heat map was generated in order to identify the chromosomal location of significantly differentially expressed genes from patient and control T cells. Clusters of significantly altered genes between SZ and C freshly isolated T-cells were found at 1p36, 1q42, 4q12, 6p22, 9q22, 10q26. 1p36, 1q42 and 6p22 are strong susceptibility loci

In order to further investigate the differences in gene expression between schizophrenia patients and controls, a heat map was generated, visualising the chromosomal locations of differentially expressed genes. Clusters of genes significantly altered between patients and controls from freshly isolated T-cells were identified at chromosomal regions 1p36, 1q42, 4q12, 6p22, 9q22, 10q26 (Figure 6). Although no clear candidate genes were identified in these regions, it is of note that 1p36, 1q42 and 6p22 are strong susceptibility loci for schizophrenia (OMIM).

## Discussion

The aim of the present study was to identify physiological differences between schizophrenia patients and healthy controls in peripheral tissue, with the hope of establishing a functionally representative tissue alternative to the brain in which to study pathophysiological mechanisms associated with this disorder.

Currently, research focuses on post mortem human brain tissue and animal models, and although considerable advances have been made using these resources, post mortem tissue can never provide the basis for dynamic functional studies and post mortem effect and suitable control matching will always be problematic [12]. Similarly, animal models are associated with major drawbacks, as until we understand the aetiology of schizophrenia, we cannot establish truly representative models of psychosis or schizophrenia. Existing models are, at best, models for hypotheses. In this study we used peripheral blood T cells as a model system for dynamic investigations into alterations in cell function with the hope of eventually relating disease-associated changes to the brain.

Schizophrenia is a clinically heterogeneous disorder with a spectrum of symptoms. Although the disorder primarily affects brain function (especially thoughts, affect, perception, social functioning and cognition), it is unknown whether other organs are affected. The tissue type and stage of disease when a given sample was collected are also likely to influence findings. Changes throughout disease progression could also significantly impede the identification of consistent pathological changes if patients are investigated at different stages of their illness. Indeed, physiological mechanisms triggering positive or negative symptoms in the early disease stages could differ significantly from chronic illness and remission. To start to address the complexity of this symptomatically defined disorder, we need to conduct studies aimed at identifying clear and consistent differences between patients and controls, ideally identifying accessible tissues in which we can detect disease-related changes. More extensive longitudinal studies will then be required to characterise the physiological changes associated with different disease states.

Our findings of lower T cell proliferative responses to anti-CD3 in schizophrenia add to previous reports of immune alterations in schizophrenia. These findings are not consistent across all studies, possibly arising from biological changes throughout the course of the disorder or reflecting the possibility that schizophrenia represents a spectrum of clinically related disorders, each with differing underlying pathologies. One of the most frequently reported immunological alterations reported in schizophrenia is an imbalance in T helper 1 (TH1) and T helper 2 (TH2) cytokine profile production with a skew towards TH2 cytokine profiles in schizophrenia patients [13], [14], suggested as reflecting diminished pro-inflammatory TH1 responses in schizophrenia. Lower numbers of CD4+ cells and hence IL-2 production have also been reported in schizophrenia [15], although Muller *et al* describe increased CD4+ cell number [16], in addition to altered plasma and serum levels of other cytokines such as IL-6 [17]. Differing proportions of leukocyte subsets with higher numbers of CD5+ B cells [18], higher proportions of naïve (CD45RA+) T cells [19] and decreased T suppressor cell activity [16] have all been reported. There have also been associations between schizophrenia and autoimmune diseases, with higher levels of autoantibodies detected in patients, including anti-cardiolipin antibodies, anti-histone, rheumatoid factor, lupus anticoagulant and antibodies to the hippocampus and septal region of the brain [20]–[24]. Kessler and Shinitzky reported platelet autoantibodies, which have been shown to inhibit dopamine uptake [25].

In the present study, we observed significantly lower proliferative responses to T cell stimulation with anti-CD3 in schizophrenia patients compared to controls, demonstrating that physiological differences can indeed be identified in peripheral tissues. There are many reasons why patient T cells may respond poorly to stimulation by proliferation. Cell functional abnormalities such as suboptimal expression of signalling molecules or genes necessary for cell cycle progression, or abnormalities with energy supply and utilisation may underlie these effects. Similarly, cell extrinsic inhibitory factors present within patients' bodies may diminish proliferative responses. Circulating stress hormones or products of oxidative stress may alter the ability of cells to respond, as may chronic T cell activation within the patients' bodies.

In the present study CD3 and TCRαβ expression were found to be comparable on patient and control T cells, demonstrating that patient cells are capable of responding to *in vitro* stimulation. Patient and healthy control T cell samples also had comparable proportions of CD4+ and CD8+ T cells, altered numbers of which could result in different rates of proliferation between patients and controls. These data suggested that there is no obvious receptor proximal abnormality which could initially prevent patient T cells from responding similarly to control cells when stimulated with anti-CD3.

Cell cycle entry marks the culmination of a complex and intertwined array of cell signals and processes, abnormalities of any one of which may be responsible for depressed proliferative responses in patient T cells. Early T cell receptor signalling was investigated by looking at global tyrosine phosphorylation events following stimulation. Patient and control T cell lysates were investigated by Western blot, using an antibody against phosphotyrosine. There were no visible differences in the levels of tyrosine phosphorylation (data not shown), representative of early Src family tyrosine kinase signalling, in patient and control T cells following stimulation, suggesting that early signalling pathways in patients are intact, and do not contribute to lower proliferative responses. Downstream of early receptor signalling, IL-2 is produced and used by T cells in an autocrine fashion in response to stimulation to drive proliferation [7]. As such, any abnormalities in the production or use of this cytokine could result in lower proliferative responses. Patients were found to produce similar amounts of IL-2 to healthy controls at a range of stimulating concentrations of anti-CD3 (Figure 4A) and expression of CD25, the specific alpha-chain of the IL-2 receptor was similar between patients and controls (Figure 4B and 4C), demonstrating that although patients have lower proliferative responses to anti-CD3, they are capable of producing and responding to IL-2. CD25 is also an indicator of activation state, with low expression on unstimulated cells which increases following stimulation. Expression and up regulation of CD25 on patient T cells demonstrates that although these cells are not proliferating as quickly as their healthy counterparts, patient T cells are capable of becoming activated and producing the cytokine IL-2 and also suggests that cell pathways involved in protein trafficking and receptor recycling are intact, with abnormalities involved in lower proliferative responses lying downstream of the bifurcation of these pathways.

Expression of CD45 isoforms was measured on patient and control T cells, both to further investigate availability of T cell receptor complex components on patient cells and as a means of assessing differences in antigen experience between patients and controls. Cazullo *et al* previously reported increased CD4+ and CD8+ CD45RA+ T cells in schizophrenia patients, suggesting higher numbers of antigen naïve T cells [19]. They suggested that exogenous factors present in the patients body stimulated expansion of the CD4+ and CD8+ CD45RA+ populations and a reduction in numbers of CD45RA- memory cells. Our data supported these finding as increased expression of CD45RA andCD45RB was found, together with a trend towards lower CD45RO expression. These data together could suggest that schizophrenia patients have higher numbers of naïve compared to antigen-experienced memory T cells. It is important to note that CD45 isoform expression is only a crude estimation of activation state. Antigen experience has been classified into four denominations; naïve cells (CD28+ CD45RA+ CCR7+ CCR6− CD62L+ CD27+ CD11a low) [26], [27], antigen experienced cells (CD45RO+ CD28− CCR7− CD11a+), central memory cells (CD45RO+ CD28+ CCR7+ CD11a +) and effecter memory cells (CD45RO+ CCR7− CD11a+) [28]. More recent evidence shows that co-expression of a range of markers including CD27, CD28, CCR5, CCR6, CCR7, CD62L and CD11a alter independently with progressive cell divisions and differentiation [26], [27], [29]. It has also been demonstrated that a subset of CD4RA+ CD27+ CD28− T cells are antigen experienced, expanded clones which respond well to re-stimulation [26]. Further studies are therefore indicated to analyse the true phenotype and function of expanded CD45RA+ T cell populations in schizophrenia to determine whether patients have increased numbers of CD45RA+ naïve T cells, or increased reverted CD45RA+ antigen experienced cells. This is however a complex and rapidly progressing field and it is challenging to assign *in vivo* function to intricate sub-phenotypes of T cell.

Our finding of increased CD45RA and CD45RB expression on patient cells clearly suggests that the patient group appear to have altered populations of antigen experienced T cells. This could be interpreted in a number of ways. Either comparable numbers of patient CD45RO+ cells are not generated from CD45RA+ cells at the same rate as in healthy control cell populations, there may be higher rates of reversion to CD45RA+ from CD45RO+ following antigen exposure, or perhaps inadequate survival factors result in deletion of generated CD45RO+ cells. Alternatively, higher numbers of CD45RA+ T cells may be generated in patients.

Regarding isoform switching of CD45RA+ naïve cells to CD45RO+ memory cells; it is very hard to imagine that schizophrenia patients are not exposed to the same infections and antigens as the rest of the general population and therefore that their CD45RA+ populations do not convert to CD45RO+ memory cells at the same rate. Cellular mechanisms involved in antigen processing and presentation and T cell responses to antigen could be less efficient in schizophrenia, reflected by our findings of lower proliferative responses to anti-CD3 stimulation. Interestingly, there are various reports in the literature that point to cellular hyporesponsiveness in schizophrenia. Riedel *et al* reported lower type IV delayed skin hypersensitivity reactions in schizophrenia patients [30], suggesting defects in cell mediated immune responses with sensitised TH-1 helper T cells and Russo *et al* importantly showed significantly lower antibody responses to vaccination with hepatitis B in psychiatric patients [31]. Together with a negative correlation of schizophrenia with chronic inflammatory diseases, such as rheumatoid arthritis [32], these data could suggest diminished T cell responses in schizophrenia, although it should be considered that chronic activation of T cells, as seen in chronic inflammatory diseases such as rheumatoid arthritis and SLE can result in hyporesponsiveness to *in vitro* stimulation [33], [34]. Higher expression of CD45RA in patient populations could instead indicate higher rates of reversion to CD45RA from CD45RO.

Despite the many reports of immune alteration in schizophrenia, there is a lack of clinical support for specific immune dysfunction in schizophrenia. Patients are not clinically immuno-compromised; there are no confirmed reports of opportunistic infections or a higher incidence of shingles or herpes reactivations, for example, although the high degree of functional redundancy in the immune system could prevent clinical immunodeficiency.

To identify deficiencies in any major functional cell pathways that may provide further evidence of cellular hyporesponsiveness, we used microarrays to identify candidate genes and pathways that may be dysregulated in schizophrenia. Functional category analysis of significantly altered transcripts using OntoExpress revealed several significantly altered pathways in each of three functional categories cell cycle, signal transduction and response to oxidative stress. Each of these processes has previously been implicated in schizophrenia and may contribute considerably to lower T cell proliferative responses. Dysregulation of transcripts involved in cell cycle from freshly isolated T cells are extremely interesting in light of the observed lower proliferative responses of patient T cells to stimulation. Transcripts were measured in freshly isolated unstimulated T cells not in cell cycle, and yet five pathways associated with cell cycle mechanisms were significantly altered in schizophrenia, suggesting there may be insufficient constitutive expression of genes and proteins necessary for normal rates of cell turnover. Alteration in the expression of genes from cell cycle categories also carries heavy implications for existing hypotheses of neurodevelopment and tissue repair, particularly as neural stem cell proliferation has been found to be decreased in schizophrenia [35].

Abnormalities in expression of genes relating to signal transduction also has huge implications for the pathophysiology of schizophrenia and its clinical presentation. Intracellular signalling is one of the most fundamental functions of a cell, crucial for most cellular processes including energy utilisation, responses to growth factors and neurotransmitter signalling. We found no deficiencies in early stimulation-induced tyrosine phosphorylation events in schizophrenia, however downstream pathways such as MAP kinase signalling and cytokine signalling may yet be affected. This certainly requires further investigation, although as signalling pathways are so complex and intertwined, identifying specific deficiencies will prove challenging.

Three pathways associated with oxidative stress and metabolism were also significantly altered in freshly isolated T cells. T cell responses would obviously be altered by any abnormalities in energy supply and utilisation, particularly energy dependent functions such as cell cycle. T cell activation is also particularly sensitive to the production of reactive oxygen species and oxidative stress, as many of the upstream signalling phosphatases contain cysteine residues in their active sites, accounting for lower proliferative responses in patients. These results were of particular interest, as these categories were also found to be significantly altered in schizophrenia in our previous study on prefrontal cortex of post mortem brains [36], as well as in several peripheral patient tissues such as liver and red blood cells [37]. In the present study, members of these functional categories showed a bias towards down regulation of expression in schizophrenia patient T cells. Overall 80% of transcripts were down regulated, mirroring alterations in protein expression from the prefrontal cortex post mortem brain study, which showed major down regulation of proteins associated with mitochondria and oxidative stress [36].

Currently there is insufficient evidence to refute or confirm the presence of specific immune dysfunction in schizophrenia and it is still unclear whether immune alterations are reflective of specific immune dysfunction, are a bystander effect of upstream regulatory mechanisms, or result from tissue pathology associated with the disorder. Immune responses are exceedingly dynamic and it is often hard to demonstrate the presence of an immune response by looking at one or two isolated aspects of the immune response at one point in time. Also, physiological inflammation is transient and may be missed if a population of patients is sampled at one time point only. Larger immunological studies need to be carried out, where several parameters can be measured and correlated for the same individual with follow up tests through different aspects of the disorder (i.e. prodromal state, psychotic episodes etc.).

The lack of specific immune dysfunction in schizophrenia is interesting in itself, given the above findings. The immune system has a high degree of functional redundancy, owing to high evolutionary pressure from the ‘arms race’ between man and microbes. It is possible that molecular pathways resulting in lower proliferative responses and T cell hyporesponsiveness are compensated for in the immune system, for example, by increased efforts of innate immunity. However the cells responsible for higher human functions such as cognitive mood may not be able to compensate for these impairments resulting in the symptoms of schizophrenia.

### Conclusion

Clear and significant physiological differences in T cell responses can be identified in schizophrenia patients, namely reduced proliferative responses to stimulation, alterations in T cell subpopulations expressing CD45 isoforms and expression of genes associated with cell cycle, cell signalling and oxidative stress and metabolism. Besides providing an easily accessible cell type in the living patient, cell biological studies can provide more in depth and robust results, as dynamic functional studies are possible and problems resulting from cell and tissue heterogeneity, as encountered when using post mortem brain homogenates are not an issue.

There is compelling evidence for cellular dysfunction in schizophrenia and the mechanisms of this and how it changes throughout disease progression need to be pinpointed in order to further our understanding of this disorder.
